# ICPi-Induced Graves' Disease with Pre-existing Autoimmune Thyroid Disorders: A Case Report and Literature Review

**DOI:** 10.2174/0118715303317264250116095028

**Published:** 2025-02-10

**Authors:** Xinpan Wang, Doudou Chen, Yun Shi, Tao Yang, Xuqin Zheng

**Affiliations:** 1 Department of Endocrinology, The First Affiliated Hospital of Nanjing Medical University (Jiangsu Province Hospital), Nanjing, China

**Keywords:** Immune checkpoint inhibitor, Graves' disease, thyroid toxicity, autoimmune disease, autoantibody, hyperthyroidism

## Abstract

**Background:**

Immune Checkpoint Inhibitor (ICPi) therapy has revolutionized cancer treatment but can lead to immune-related adverse events (irAE), including thyroid dysfunction. The impact of ICPi on patients with pre-existing autoimmune thyroid diseases (PATD), particularly the development of Graves' disease, remains poorly understood.

**Case Presentation:**

We provide the first complete case of Graves' disease with ICPi therapy in a patient who already had Hashimoto's thyroiditis. The patient, a 52-year-old male, was diagnosed with lung adenocarcinoma and received Atezolizumab. Clinical evaluation revealed hyperthyroidism, confirmed by elevated thyroid hormones and autoantibodies (TRAb and TSAb). The patient was managed with methimazole and demonstrated a transient hyperthyroid phase followed by persistent hypothyroidism. Only 16 confirmed cases of Graves' disease induced by ICPi were reported. We conducted a review to investigate the clinical characteristics, risk factors, and prognosis trends associated with ICPi-induced Graves disease in PTAD patients. Additionally, changes in thyroid function and autoantibodies during and after ICPi treatment are examined.

**Conclusion:**

This case underscores the importance of monitoring thyroid function and autoantibodies in patients with PATD undergoing ICPi therapy. The findings suggest distinct differences in the humoral immune response between ICPi-induced and spontaneous Graves' disease, necessitating further research into autoantibody dynamics and their relationship with cellular immunity in these patients.

## 1 INTRODUCTION

Immune checkpoint inhibitors, as one of the common immunotherapy methods for tumors, shift the therapeutic target from the cancer to the immune system, but they can be accompanied by immune-related adverse events, affecting the thyroid gland, pituitary gland, pancreas, adrenal gland, and parathyroid gland [1-3]. The exact mechanism of how ICPi causes autoimmune endocrine gland dysfunction is still unclear [4]. Due to the concern about potential adverse reactions and expected curative effects, patients with pre-existing autoimmune disease (PAD), especially autoimmune endocrine disease, are usually excluded from clinical trials involving ICPi for tumors [5-8]. As the incidence of autoimmune diseases continues to increase worldwide, physicians will face the problem of whether to administer ICPi to PAD patients [9].

As the most common irAE, the incidence of thyroid dysfunction in non-PAD patients is about 3% -8%, mainly manifested as hypothyroidism and a small part is destructive thyroiditis. At the same time, Graves' disease is rare [10, 11]. We present a rare case of Atezolizumab-induced Graves disease in a patient with pre-existing autoimmune thyroid disease. This case study highlights the long-term trend of four thyroid autoantibodies, which may explain the changes in thyroid immune balance and provide insight into potential biomarkers for identifying high-risk patients with Graves' disease induced by ICPi.

### 2 CASE PRESENTATION

We received a 52-year-old male patient who underwent right bullae resection and repair because of spontaneous pneumothorax. Postoperative pathology revealed poorly differentiated adenocarcinoma of the bullae. After radical resection of lung adenocarcinoma, the patient received “Pemetrexed 800 mg + Carboplatin 450 mg each time” chemotherapy for 4 cycles. After completing the chemotherapy, the patient started immune checkpoint inhibitors. The patient suffered from palpitation, fear of heat, excessive sweating, and weight loss two weeks after the first infusion of Atezolizumab (PD-1 inhibitor, 1200 mg) for lung adenocarcinoma. The patient had a history of Hashimoto's thyroiditis with normal thyroid function for one year before ICPi therapy and didn’t take thyroid-related medications such as levothyroxine, during which thyroid condition was monitored continuously. In the stage of Hashimoto's thyroiditis (87-150 days before ICPi), the fluctuation of thyroid stimulating hormone (TSH) was 1.45-2.48 mIU/L (normal range 0.27-4.2), that of FT3 was 4.07~5.15 pmol/L (normal range 3.1-6.8) and that of FT4 was 15.8-18.6 pmol/L (normal range 12-22). Thyroglobulin autoantibody (TgAb) was positive, fluctuating in the range of 144.8-374.2 IU/ml (normal range 0-115), while thyroid peroxidase autoantibody (TPOAb) (28.5 IU/ml, normal range 0-34), thyroid stimulating hormone receptor autoantibody (TRAb) (<0.8IU/L, normal range 0-1.5) and thyroid stimulating antibody (TSAb)(0.1 IU/L, normal range 0-0.55) were all negative. Additionally, the patient displayed diffuse thyroid changes on ultrasound, consistent with Hashimoto's thyroiditis.

**Fig. (1):**
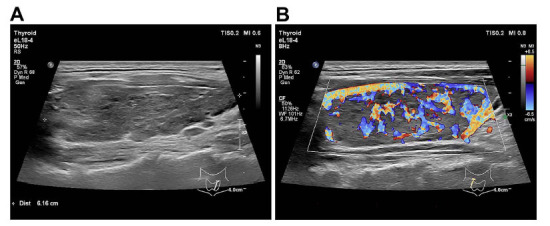
Thyroid ultrasonography. (**A**). Slight swelling in lobe. (**B**). Rich blood flow in parenchyma.

On admission, the physical examination revealed tachycardia, tremors in the fingers, and painless grade II thyroid enlargement without any signs of Graves' ophthalmopathy. Hyperthyroidism deteriorated rapidly within a few days: FT3 increased from 27.91 to more than 50 pmol/L (normal range 3.1 to 6.8), FT4 increased from 95.29 to more than 100 pmol/L (normal range 12 to 22), and TSH decreased from 0.015 to 0.009 mU/L (normal range 0.27 to 4.2). TRAb (9.45 IU/L, normal range 0-1.5), TSAb (4.45 IU/L, normal range 0-0.55), TPOAb (127 IU/ml, normal range 0-34), and TgAb (557 IU/ml, normal range 0-115) were all positive. Thyroid ultrasound revealed slightly plump thyroid glands on both sides (right lobe 58*24*23 mm, left lobe 62*26*22 mm) and abundant blood flow signals within the thyroid (Figs. **1A** and **1B**). Regrettably, the patient did not undergo a thyroid scintigraphy test, such as a radioiodine or technetium scintigraphy. We screened other important endocrine-related hormones, including sex hormones, cortisol, adreno-cortico-tropic hormone (ACTH), growth hormone, and parathyroid hormone, which were all within normal ranges. The results of the Oral Glucose Tolerance Test showed impaired glucose tolerance, with a fasting glucose level of 5.52 mmol/L and a 120-minute glucose level of 9.1 mmol/L. In contrast, C peptide and insulin levels were normal.

**Fig. (2):**
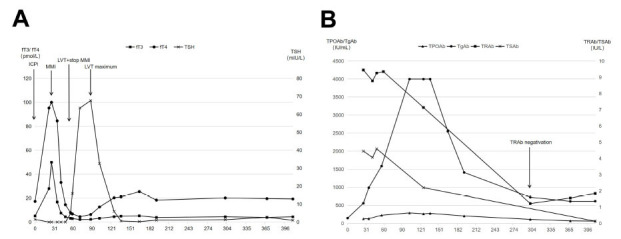
Evolution of thyroid dysfunction. (**A**). Thyroid hormone. (**B**). Thyroid autoantibodies. **Abbreviations:** FT3: free triiodothyronine; FT4: free thyroxine; TSH: thyroid stimulating hormone; ICPi: immune checkpoint inhibitor; MMI: methimazole; LVT: levothyroxine; TRAb: thyroid stimulating hormone receptor autoantibody; TPOAb: thyroid peroxidase autoantibody; TgAb: thyroglobulin autoantibody; TSAb: thyroid stimulating hormone receptor stimulating antibody.

Based on clinical presentation, ultrasound findings, thyroid function tests, and antibody levels, the patient was diagnosed with Graves' disease. The patient discontinued ICPi treatment and started taking methimazole 30 mg/day. Thyroid function was similar after one week of anti-thyroid drug therapy (FT3 48.6 pmol/L, FT4 >100 pmol/L). Two weeks after initiating methimazole, the patient developed hepatic impairment with an elevated aspartate aminotransferase of 142.4 U/L (normal range 15-40). At the same time, considering the rapid decline of thyroid function, the dosage of methimazole was reduced from 30 mg to 10 mg. In the third week of methimazole treatment, thyroid function further deteriorated, and methimazole was reduced to 5 mg. In the 4th week after the start of methimazole, the thyroid function indicated subclinical hypothyroidism with FT3 at 3.18 pmol/L, FT4 at 7.88 pmol/L, and TSH at 4.56 mIU/L, leading to the withdrawal of methimazole. Only three days after stopping methimazole, the thyroid function showed a rapidly progressive decline, and it reached a trough one month after anti-thyroid drugs termination (FT3 2.3 pmol/L, FT4 6.3 pmol/L, TSH 67.65 mIU/L) (Fig. **2A**). At the same time, TPOAb and TgAb levels peaked at 289.2 IU/ml and >4,000 IU/ml, respectively. The patient received a maximum dose of 125 ug/day of levothyroxine as a hormone supplement. During the following 12 months of observation, the patient was followed up approximately every 1-3 months. The patient maintained normal thyroid function with levothyroxine (75 ug/day) and there was a slow decline in antibody titers for TPOAb, TgAb, TRAb, and TSAb. However, until the end of the observation period, the patient was still positive for above thyroid autoantibodies except TSAb, and the TgAb level was significantly higher than that before the immunotherapy (Fig. **2B**). The patient demonstrated good compliance, strictly adhering to the prescribed medication and attending regular follow-ups. The monitoring frequency and follow-up time points are shown in Fig. (**2**). In addition, serum levels of FT3, FT4, TSH, TPOAb, TgAb, and TRAb were measured using the modular analytics E170 fully automated electrochemiluminescence immunoassay system and matching reagent kits (Roche Diagnostics, Germany). We performed thyroid ultrasound examinations on the patient using the Siemens color Doppler ultrasound diagnostic instrument (Germany) with a probe frequency of 5-15 Hz.

## 3 DISCUSSION

Most cases of ICPi-associated thyroid toxicity are transient changes caused by destructive thyroiditis, which are typically followed by persistent hypothyroidism. Graves' disease is rarely observed [10, 12]. Graves' disease is an autoimmune thyroid disorder associated with multiple genetic loci, and its exact mechanisms remain unclear [13-15]. Potential triggers for secondary Graves' disease may include medications (such as amiodarone), bacterial or viral infections, and pregnancy [16, 17]. Considering the patient's medical history, medication history, and endocrine test results, it is most likely that ICPi induced the patient's Graves' disease, and the influence of genetic susceptibility and other complications remains undetermined. In this case, due to liver function impairment and rapid improvement in thyroid function, the patient received a relatively short course of antithyroid drug (ATD) therapy. Unlike the typical 1-2 years of ATD treatment required for spontaneous Graves' disease [16], the treatment duration for ICPi-related Graves' disease may have different characteristics. This short-term treatment could be related to the specific mechanisms of ICPi-induced Graves' disease, and the rapid deterioration and recovery of thyroid function may suggest a different disease course.

A systematic exploration of the Medline database was conducted using the keywords: “immune checkpoint inhibitor” and “Graves' disease”. A comprehensive search strategy was employed, incorporating Boolean operators (AND, OR) to combine terms effectively. The search was limited to articles published in peer-reviewed journals, focusing specifically on studies with human subjects and written in English to ensure relevance and accessibility. Article types included randomized controlled trials, cohort and case-control studies, case reports, meta-analyses, and systematic reviews. Studies written in languages other than English were excluded.

Additionally, reference lists of identified articles were reviewed for additional relevant studies. Data extraction concentrated on study design, patient demographics, clinical outcomes, and any reported adverse effects related to immune checkpoint inhibitors. As of May 2024, there have been 16 reported cases of ICPi-induced Graves' disease (Table **1**) [18-29], excluding cases of thyroid eye disease (TED)-like orbital infection syndrome without hyperthyroidism. None of the patients had a history of autoimmune thyroid disease, and none developed Graves ophthalmopathy following ICPi treatment. Among patients with ICPi-induced Graves' disease, 67% were male, and 25% developed secondary hypothyroidism after initial hyperthyroidism, which showed great heterogeneity in the course and clinical characteristics compared to spontaneous classic Graves' disease (Table **2**).

Previous studies have shown that having positive baseline levels of TgAb or TPOAb increases the risk of developing immune checkpoint inhibitor (ICPi)-induced thyroid autoimmune disease [30-34]. In our case, the patient with pre-existing thyroiditis was positive for TgAb at baseline, which may represent a certain tendency. In addition, the increase of TPOAb and TgAb began in the thyroid toxicity phase. TgAb, TPOAb, and thyroid function showed consistent trends during the hypothyroidism phase. The patient experienced the most severe hypothyroidism, during which their TSH levels reached 32.59 mU/L (normal range 0.27 to 4.2), FT3 levels reached 19 pmol/L (normal range 3.1 to 6.8), and FT4 levels reached 12.65 pmol/L (normal range 12 to 22), requiring treatment with levothyroxine at a dosage of 125 ug/day. The peak levels of TPOAb and TgAb were observed during this phase. Similar to destructive thyroiditis, the changes in TPOAb and TgAb seem to be an antibody response to the release of thyroid antigens after injury [35, 36]. However, explaining the transition from negative to significantly positive for TRAb and TSAb poses a challenge, as they are typically used as molecular markers to differentiate between destructive thyroiditis and Graves' disease [13, 37, 38].

Among the reviewed cases of ICPi-induced Graves' disease, 14 patients had positive TRAb or TSAb at the time of diagnosis. In two cases, the follow-up showed that the normalization of TRAb occurred significantly later than the normalization of thyroid function [23, 26]. This is consistent with our case, where TRAb remained positive even after hyperthyroidism turned into hypothyroidism. The review did not provide a continuous measurement record of TSAb in ICPi-related Graves' disease. Our study showed that the trend of TSAb was similar to that of TRAb, where although the antibody levels decreased slowly, they remained positive for an extended period during hypothyroidism. This is an intriguing finding, as TRAb and TSAb are generally not stable for an extended period during hypothyroidism, except in cases of drug-induced hypothyroidism or during the recovery phase after radionuclide therapy [39-41].

**Fig. (3):**
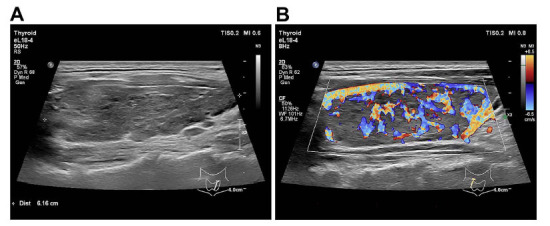
Potential mechanism of immune-related thyroid adverse events and antibodies evolution.

Several studies have shown that the key for ICPi to induce autoimmune diseases, including Graves' disease, is to inhibit the negative stimulation molecules on the T cell surface, disrupt the T cell-related immune homeostasis, and reactivate the exhausted CD4^+^ and CD8^+^T cells [10, 42-44]. According to the humoral immunity theory of spontaneous Graves' disease (unrelated to ICPi), germinal center B cells proliferate and differentiate under the action of activated T cells and rapidly secrete large amounts of TRAb driven by TSHR antigen, leading to thyroid toxicity [45, 46]. There are several possible explanations for the changes in TSAb and TRAb in the patient of the case (Fig. **3**): (1) TSAb is the dominant antibody in hyperthyroidism. After the external stimulation of ICPi is stopped, B cell secretion is decreased, and the stimulating antibody TSAb falls off from TSHR and spontaneously decays. However, due to its long half-life, a significant amount of free TSAb remains in the body during hypothyroidism [3, 7, 47, 48]. (2) The stimulating antibody TSAb and the inhibitory antibody TBAb, which are subtypes of TRAb, coexist in the body for an extended period. In the early stage, TSAb reaches a high affinity for maturation and competitively occupies TSHR. In contrast, in the late stage, the inhibitory antibody TBAb surpasses in affinity, leading to the tilt of hypothyroidism [39, 40]. (3) Although PD-1 inhibitor can directly induce cytotoxic lymphocyte (CTL) activation of CD8^+^ and destructive TPOAb and TgAb production, the humoral immune changes of TgAb and TPOAb are slower than those of CD4^+^T cell-induced TRAb and TSAb. In the early stage of ICPi-induced immune imbalance, the CD4^+^T cell-TRAB/TSAB axis dominates, which may explain why the bidirectional change of thyroid induced by ICPi is always hyperthyroidism to hypothyroidism rather than hypothyroidism to hyperthyroidism [45, 49, 50].

Many studies have confirmed the role of T cells in the immune function of thyroid autoimmune diseases induced by ICPi. Currently, there is a lack of research and explanation on humoral immunity, particularly regarding changes in the antibody titer of the TRAb subtype. More prospective data is required in the future to establish the relationship between changes in the autoantibody titer of Graves' disease induced by ICPi and cellular immunity. These potential biomarkers could help understand the similarities and differences between Graves' disease induced by immune checkpoint inhibitors and spontaneous classical Graves' disease and assist in identifying high-risk patients with Graves' disease induced by ICPi.

## CONCLUSION

The case contributes to our understanding of the impact of ICPi therapy on patients with pre-existing autoimmune thyroid diseases, particularly in relation to the development of Graves' disease. The findings emphasize the need for vigilant monitoring of thyroid function and autoantibodies in these patients during ICPi treatment. The proposed humoral immune mechanism provides a basis for further research and potential biomarkers to identify high-risk patients. Overall, this study aims to improve our knowledge of ICPi-associated thyroid dysfunction and assist healthcare professionals in providing effective and personalized care for patients receiving ICPi therapy.

## AUTHORS’ CONTRIBUTIONS

X.W. drafted the manuscript and conducted the literature review. D. C. and Y.S. contributed to data collection and analysis. T.Y. and X.Z. supervised the study, provided critical revisions, and finalized the manuscript. All authors have read and approved the final version of the manuscript.

## Figures and Tables

**Table 1:** Review of the literature: case reports of Graves’ hyperthyroidism after ICPi treatment.

First Author, Year	ICPi	Sex, Age	Baseline Thyroid Condition (Function/ antibody)	Hyperthyroidism		Spontaneous Hypothyroidism	Euthyroidism	TRAb /TPOAb /TgAb Normalization	Final Thyroid Function	Observation Period
				Cycle/Time*	Antibody (TRAb/ TPOAb/ TgAb)					
Azmat, 2016, [18]	anti-CTLA-4 (Ipilimumab)	M,67	Nor, na	2,1 m	Pos, na, na	No	na	na, na, na	Hypothyroidism	na
Filette, 2016, [19]	anti-PD-1 (Pembrolizumab)	na, na	na, na	na, na	Pos, na, na	Yes	na	na, na, na	Hypothyroidism	na
Gan, 2017, [20]	anti-CTLA-4 (Tremelimumab)	M,55	Nor, na	20,8 y	Pos, Pos, na	No	8w	na, na, na	Euthyroidism	18m
Narayen, 2019, [21]	anti-PD-1 (Pembrolizumab)	F,37	na, na	2,na	Pos, na, na	No	na	na, na, na	na	na
Brancatella, 2019, [22]	anti-PD-1 (Nivolumab)	M,51	Nor, TPOAb and TgAb Neg	4,56 d	Neg, Neg, Neg	No	60d	Remain Neg, remain Neg, remain Neg	Euthyroidism	6m
Yajima, 2019, [23]	anti-PD-1 (Pembrolizumab)	M,61	Subclinical hyperthyroidism, TRAb Neg	5,102 d	Pos, Neg, Neg	No	51 d	135 d, na, na	Hypothyroidism	135d
Iadarola, 2019, [24]	anti-PD-1 (Nivolumab)	M,66	Nor, TPOAb and TgAb Neg	2,2 w	Neg, Neg, Neg	No	7m	Remain Neg, remain Neg, remain Neg	Euthyroidism	9m
Yamada, 2020, [25]	anti-PD-1 (Nivolumab)	M,66	Nor, all Neg	2,43 d	Pos, Neg, Neg	No	No euthyroid	Remain Pos, remain Neg, remain Neg	Subclinical hyperthyroidism	96d
Kurihara, 2020, [26]	anti-PD-1 (Nivolumab)	M,48	Nor, na	6,127 d	Pos, Neg, Neg	No	75d	213 d, remain Neg, remain Neg	Subclinical hypothyroidism	450d
Peiffert, 2021, [27]	anti-PD-1 (Pembrolizumab)	F,69	Nor, na	9,41 w	Pos, Neg, na	No	11w	6 m, remain Neg, na	Euthyroidism	57w
Peiffert, 2021, [27]	anti-PD-L1 (Durvalumab) and anti-CTLA-4 (Tremelimumab)	F,60	Nor, na	1,2 w	Pos, Pos, na	No	na	na, na, na	Hyperthyroidism	30w
Peiffert, 2021, [27]	anti-PD-1 (Pembrolizumab)	M,60	Nor, na	1,3 w	Pos, Pos, na	Yes	13w	na, na, na	Hypothyroidism	48w
Peiffert, 2021, [27]	anti-PD-1 (Nivolumab) and anti-CTLA-4 (Ipilimumab)	F,44	Nor, na	1,2 w	Pos, na, na	Yes	na	na, na, na	Low FT3/FT4 and normal TSH	13w
Peiffert, 2021, [27]	anti-PD-1 (Nivolumab)	M,76	Nor, na	2,4 w	Pos, Neg, na	Yes	na	na, remain Neg, na	Hypothyroidism	143w
Reddy, 2021, [28]	anti-PD-1 (Pembrolizumab)	F,57	Nor, na	na,15 m	Pos, Neg, Neg	No	na	na	na	2y
Alqaisi, 2023, [29]	anti-PD-1 (Pembrolizumab)	M,50	na, na	1,5 w	Pos, Pos, Pos	No	na	na, na, na	na	1m

**Note:** Time begins with thyrotoxicosis after ICPi (except where otherwise specified). **Abbreviations:** ICPi: immune checkpoint inhibitor; TRAb: thyroid stimulating hormone receptor autoantibody; TPOAb: thyroid peroxidase autoantibody; TgAb: thyroglobulin autoantibody; F: female; M: male; d: day; w: week; m: month; y: year; neg: negative; pos: positive; anti-PD-1: anti-programmed cell death 1 protein antibodies; anti-PD-L1: anti-programmed cell death ligand-1 antibodies; anti-CTLA-4: anti-cytotoxic T-lymphocytic-associated antigen-4 antibodies; FT3: free triiodothyronine; FT4: free thyroxine; TSH: thyroid stimulating hormone. *Cycle/Time is calculated from initial use of ICPi.

**Table 2:** Spontaneous classical Graves' disease and ICPi-induced Graves' disease: differential diagnosis.

-	Spontaneous Classical Graves' Disease	ICPi-induced Graves' Disease
Cause	Unknown, possibly due to genetics and environment	ICPi
Gender advantage	Female	Male
Biphasic changes of thyroid function	Rare	Common
Thyroid ophthalmopathy	Often present with hyperthyroidism	No known occurrence of hyperthyroidism and eye disease coexisting
TRAb	Positive, normalizes with remission	Positive in hyperthyroidism, remains positive for an extended period during hypothyroidism
TgAb and TPOAb	+/-	+/-
Treatment	Anti-thyroid drugs regularly	Dynamic adjustment of antithyroid drugs and hormone supplementation in hypothyroidism

**Abbreviations:** ICPi: immune checkpoint inhibitor.

## Data Availability

The data and supportive information are available within the article.

## LIST OF ABBREVIATIONS

ICPi: Immune Checkpoint Inhibitor
irAE: Immune-related Adverse Events
PATD: Pre-existing Autoimmune Thyroid Diseases

## STANDARDS FOR REPORTING

CARE guidelines and methodology were followed to conduct the study.
